# Normal variation in fronto-occipital circuitry and cerebellar structure with an autism-associated polymorphism of CNTNAP2

**DOI:** 10.1016/j.neuroimage.2010.02.018

**Published:** 2010-11-15

**Authors:** Geoffrey C.Y. Tan, Thomas F. Doke, John Ashburner, Nicholas W. Wood, Richard S.J. Frackowiak

**Affiliations:** a Wellcome Trust Centre for Neuroimaging, Institute of Neurology, University College London, UK; b Department of Molecular Neuroscience, Institute of Neurology, University College London, UK; c Service de Neurologie, Centre Hospitalier Universitaire Vaudois, 1011-Lausanne, Switzerland

**Keywords:** Magnetic resonance imaging (MRI), Voxel-based morphometry (VBM), Diffusion tensor imaging (DTI), CNTNAP2, Autism, Endophenotype

## Abstract

Recent genetic studies have implicated a number of candidate genes in the pathogenesis of Autism Spectrum Disorder (ASD). Polymorphisms of CNTNAP2 (contactin-associated like protein-2), a member of the neurexin family, have already been implicated as a susceptibility gene for autism by at least 3 separate studies. We investigated variation in white and grey matter morphology using structural MRI and diffusion tensor imaging. We compared volumetric differences in white and grey matter and fractional anisotropy values in control subjects characterised by genotype at rs7794745, a single nucleotide polymorphism in CNTNAP2. Homozygotes for the risk allele showed significant reductions in grey and white matter volume and fractional anisotropy in several regions that have already been implicated in ASD, including the cerebellum, fusiform gyrus, occipital and frontal cortices. Male homozygotes for the risk alleles showed greater reductions in grey matter in the right frontal pole and in FA in the right rostral fronto-occipital fasciculus compared to their female counterparts who showed greater reductions in FA of the anterior thalamic radiation. Thus a risk allele for autism results in significant cerebral morphological variation, despite the absence of overt symptoms or behavioural abnormalities. The results are consistent with accumulating evidence of CNTNAP2's function in neuronal development. The finding suggests the possibility that the heterogeneous manifestations of ASD can be aetiologically characterised into distinct subtypes through genetic-morphological analysis.

## Introduction

Autism is a complex developmental disorder typically diagnosed on the basis of early abnormalities in social interaction, language and communication impairments and the presence of repetitive behaviours and restricted interests (Lord et al., 1994). However like many other neurological and psychiatric conditions, autism is both clinically and genetically heterogeneous (Bill and Geschwind, 2009). Due to the difficulty in accurately subtyping autism, it has been clinically grouped with “pervasive developmental disorder not otherwise specified (PDD-NOS)” and the less severe Asperger's syndrome as Autistic Spectrum Disorder (ASD). Additionally, a number of other developmental disorders such as Fragile-X (Hernandez et al., 2009) and Turner's (Skuse, 2000; Lawrence et al., 2003) syndromes demonstrate autistic manifestations. Genetic studies show equal heterogeneity in regions containing mutations and de novo copy number variants associated with the disorder (Sebat et al., 2007). This variability has hampered genetic analysis and introduced potential inconsistencies into autism research because various populations and aetiologies have been investigated under the same umbrella.

This predicament could be simplified by the endophenotype concept, popular in the recent genetic neuroimaging literature. This engenders an approach that investigates a genetically complex disease through its component internal phenotypes. Unfortunately the approach has been unsuccessful from the perspective of gene discovery. Few if any genes first found by association with an endophenotype have been shown to confer sizeable risk for the disease of interest. More commonly, studies have investigated neural and behavioural correlates with polymorphisms already associated with disease in order to understand the influences of a gene on human variation at the systems level. While often taken in isolation, implicated neural systems and traits can be further used to investigate disease subtypes and motivate specific clinical interventions.

Autism-associated candidate polymorphisms are well suited for this approach. Major criteria for the definition of a trait as an endophenotype include heritability, cosegregation with disease features and its occurrence in non-affected family members at a higher rate than the normal population. Both autism, with a heritability of over 70% (Bailey et al., 1995), and brain morphology, with volumes of the whole brain and frontal regions being over 90% heritable (Toga and Thompson, 2005), are largely genetic. Additionally, it has often been suggested that autism constitutes a spectrum or continuum that extends from the diagnosis of full-blown autism to mild behavioural impairment that is compensated before the onset of adulthood (Wing, 1988). While still debated, this theory has been corroborated by self-reported characteristics in an autistic-spectrum quotient (Baron-Cohen et al., 2001) and by the ability to perform tests of theory of mind (Baron-Cohen et al., 1996). The argument for an autistic continuum is further compounded by the findings of a study performed on the apparently normal parents of autistic children. The study found significant morphological differences in many regions of the brain, compared to age and sex matched controls, in cortical regions implicated by results from patients with manifest ASD (Peterson et al., 2006). The question that remains is whether this differential, genetically-associated anatomical endophenotype is associated with behavioural or cognitive traits that are shared qualitatively with ASD patients.

ASD seems suitable for an endophenotypic method of investigation as it is improbable that the range of clinical presentation can be explained by an individual cognitive or anatomical abnormality caused by aberrant expression of a single gene (Viding and Blakemore, 2007). By fractionating the complete autistic phenotype into component parts a link could be established between genotypic variation and the integrity of brain areas implicated by any relevant impaired cognitive process (Gottesman and Gould, 2003). One such example is provided by the HOXA1 A218G polymorphism, which has been linked to increased head circumference in autistics and is the first gene to be associated with cranial morphology (Conciatori et al., 2004). Contactin (CNTN)-associated protein-2 (CNTNAP2) is a gene suitable for an endophenotypic approach as polymorphisms have been implicated in a variety of associated neuropsychiatric conditions, including ASD, epilepsy and schizophrenia (Strauss et al., 2006; Friedman et al., 2007; Burbach and van der Zwaag, 2009). Recently four independent studies found several CNTNAP2 polymorphisms associated with increased susceptibility for ASD (Alarcón et al., 2008; Arking et al., 2008; Bakkaloglu et al., 2008; Rossi et al., 2008). In addition, there is a functional genetic link between a CNTNAP2 polymorphism and FOXP2 in non-autistics with specific language impairment (Vernes et al., 2008). This finding indicates that certain phenotypic traits, or endophenotypes, transcend conventional nosological diagnostic boundaries.

Molecular studies have identified a possible role for CNTNAP2 in the brain. CNTNAP2 is found on chromosome 7q35–36.1 (Alarcón et al., 2002) and encodes contactin-associated protein-like 2 (Caspr2), a single-pass trans-membrane protein that is a member of the neurexin superfamily (Baumgartner et al., 1996). Caspr2 is co-localised and associated physically with Shaker-like voltage-activated K1 channels (Kv1) in the juxtaparanodal region of myelinated axons (Poliak et al., 1999). A study in mice involving the deletion of CNTNAP2 by gene-targeting suggested that the protein is required to maintain the function of Kv1 channels (Poliak et al., 2003). It is thought that complexes of Caspr2 and contactin are involved in interactions between neuronal axons and glia (Corfas et al., 2004). Interactions have already been found between neurexins and other genes already implicated in ASD (Jamain et al., 2003; Comoletti et al., 2004). In situ hybridisation of human foetal brains has revealed that a CNTNAP2 transcript is confined to developing frontotemporal-subcortical circuits known to be critical for executive function (Alarcón et al., 2008). It has been postulated that Caspr2 has a role in the development of the cortex and may be involved in interactions between cells during migration of the neuroblast and subsequent laminar organisation (Strauss et al., 2006).

On account of CNTNAP2's localisation and axon-related function we were particularly interested in studying white matter and employed two types of structural MRI techniques; voxel-based comparisons of fractional anisotropy (FA) using diffusion tensor imaging (DTI) (Basser et al., 1994) and voxel-based morphometry (VBM) (Ashburner and Friston, 2000). While DTI was used to study the architecture of cerebral white matter, we chose to carry out VBM on both grey and white matter because autism has been associated with increased cortical grey to white matter ratio and decreased volume of those compartments beyond childhood (Courchesne et al., 2001; Acosta and Pearl, 2004). Several studies with VBM and DTI have already been performed on various autistic populations with a wide range of results. Both increases and decreases of grey and white matter volumes and FA have been observed. The variability of these results is unsurprising given that the studied populations differed both in age and in autistic diagnosis (Eigsti and Shapiro, 2003). However certain regions have been consistently implicated by VBM studies, for example the cerebellum (Abell et al., 1999; Boddaert et al., 2004; Ke et al., 2008; Stanfield et al., 2008).

Our approach was to investigate whether differences in the polymorphism of rs7794745 CNTNAP2 among normal people conferred differences in brain structure (Arking et al., 2008). Other studies implicating CNTNAP in ASD have investigated its associations with specific facets of ASD (Alarcón et al., 2008; Vernes et al., 2008). We decided to investigate this particular polymorphism because it is associated solely with a diagnosis of autism that fulfills strict criteria and because the result has been replicated across two family-based cohorts. The authors report a significantly increased risk for autism in male homozygotes for the risk allele but not in females or male carriers.

Therefore we hypothesized significant differences in brain structure in subjects homozygous for the CNTNAP2 rs7794745 risk allele (TT) compared to heterozygous carriers for the risk allele (AT) and those homozygous for the normal polymorphism (AA) in people without ASD. Furthermore we hypothesized that differential regional brain morphology would be detected in areas implicated in a particular cognitive ability, or endophenotype, that is dysfunctional in ASD. Specifically we supposed that, on the basis of studies associating other CNTNAP2 polymorphisms to language deficits (Alarcón et al., 2008; Vernes et al., 2008) structural differences would be found in language areas. As ASD has a four-fold higher prevalence in males (Bryson, 1996) and males with CNTNAP2 risk polymorphisms are predominantly responsible for the association between CNTNAP2 and ASD (Alarcón et al., 2008; Arking et al., 2008), we also hypothesized a gender difference in the structural variation conferred by the CNTNAP2 rs7794745 risk polymorphism.

## Methods

### Participants

314 healthy volunteers were genotyped from a sample of 384 screened for neurological conditions and past psychiatric disorders including ASD using the Mini International Neuropsychiatric Inventory (Sheehan et al., 1998). They provided blood samples for genotyping and underwent T1-weighted anatomical MRI brain scans. 114 of this cohort also had DTI scans. Participants had no history of spinal or brain surgery, or of brain trauma and all had a normal MRI brain scan. The study was approved by the local ethics committee and written informed consent was obtained from all participants.

### Genotyping

DNA was extracted from blood samples and genotyped by PCR-RFLP. Primers were designed on Primer3 software (Rozen and Skaletsky, 2000) from a DNA region 500 bp flanking rs7794745, found and blasted by electronic PCR on the UCSC genome browser NCBI build 36.1 (Karolchik et al., 2008). The restriction enzyme Tsp509I (New England Biolabs) was selected as the enzyme with differential cleavage at rs7794745 on NEBcutter (Vincze et al., 2003) such that a sense strand T allele produces a fragment of 214 bp and 57 bp (not fluorescent), and an A allele produces a fragment of 271 bp.

Forward: 5′ HEX- ggcccttgcatatagttcca- 3′

Backward: 5′- ccaacagtgccttgtgtca- 3′

Genotyping was performed through polymerase chain reactions (PCR) followed by restriction digest and subsequent capillary electrophoresis. PCR with Taq polymerase (Molzym) involved initial denaturation at 94 °C for 5 min, followed by 35 cycles of denaturation at 94 °C for 15 s, annealing at 60 °C for 30 s and elongation at 72 °C for 45 s, followed by 72 °C for 7 min. Restriction digest was performed using 1 U of Tsp509I, and 2 μL PCR product in 4 μL total volume at 65 °C for 5 h. This was heat denatured to single-stranded fragments in formamide and run with a ROX500 ladder on a 3730xl DNA Analyser (Applied Biosystems). Individual genotypes were called according to peak size on GeneMapper software version 4.0 by 2 independent raters into those homozygous for the T allele, heterozygous for the T and A alleles and homozygous for the A allele.

### Image acquisition

MRI was performed on a Siemens Sonata scanner operating at 1.5 T and a Siemens Allegra scanner operating at 3.0 T (Erlangen, Germany). For VBM, a three-dimensional structural MRI scan was acquired from each subject using a T1-weighted MDEFT sequence (176 slices, 1 mm isotropic, no interslice gap, sagittal acquisition, FoV 224 × 256 mm, matrix 224 × 256, fat saturation) (Deichmann et al., 2004).

For DTI, the DW images were acquired by applying gradients in 61 directions with a *b*-value of 1000 s/mm^2^ as well as 7 with a low *b*-value of 100 s/mm^2^. The two sets of images were then processed to generate statistical parametric maps for grey matter volume, white matter volume and fractional anisotropy,

### DTI preprocessing

Images were processed using FSL software (FMRIB Software Library, FMRIB, Oxford, UK) (Smith et al., 2004). The 7 low b100 images were co-registered to create a mean image to which all 68 images DW images were co-registered by an affine transform and eddy-corrected using FLIRT and FDT (FMRIB Linear Image Registration and Diffusion Toolbox). A brain mask was created from the mean b100 image and FDT was used to fit a tensor model to compute FA, MD, axial diffusivity and radial diffusivity. Preparation for FA VBM was performed using a modification of the tract-based spatial statistics (TBSS) procedure (Behrens et al., 2003, Woolrich et al., 2009). Briefly, TBSS was used to run the initial preprocessing and nonlinear registration to the MNI-DTI template, also known as the FMRIB50_FA image in standard space (Smith et al., 2006). A mean FA and skeleton images derived from the FMRIB50_FA images were also created. Skeleton images were thresholded at 0.2 and used as an extrinsic mask in subsequent FA VBM analysis with SPM5, using smoothed warped FA images. These procedures allowed a direct comparison between WM VBM and FA VBM results.

### VBM preprocessing

VBM was performed on grey and white matter volumes and FA maps with statistical parametric mapping software (SPM5; Wellcome Trust Centre for Neuroimaging, London, UK, http://www.fil.ion.ucl.ac.uk/spm/) run on Matlab version 7.0. Grey and white matter images were created in native space for each individual using unified segmentation to partition each T1 image into tissue classes for grey matter, white matter, CSF, and non-brain voxels (Ashburner and Friston, 2005). The resulting segmented tissue probability images were used to generate a nonlinear warp to a population template generated from the complete dataset using DARTEL (Diffeomorphic Anatomical Registration Through Exponentiated Lie algebra) for more precise inter-subject alignment, increasing both sensitivity to and localization of anatomical differences (Ashburner, 2007). The MNI single subject T1-weighted atlas was also warped to the population template and a composite deformation of each subject into MNI space was used to generate modulated grey and white matter images, which were then smoothed by an 8 mm FWHM Gaussian kernel.

### Statistical analysis

In the analysis, we examined the main effect of genotype and interactions with sex by creating whole brain statistical parametric maps (SPMs) for regional grey and white matter volume, as well as FA. In the general linear model, sex, genotype and scanner acquisition were factors in the analysis with nuisance covariates for age and a global measure of either volume or FA respectively. Variables were orthogonalised using a Gram–Schmidt process implemented in SPM. We tested for both increases and decreases in grey and white matter volume and FA between TT, at risk homozygotes, and AT/AA, heterozygotes and major allele homozygotes. We performed sex interaction analyses after the GM, WM and FA analyses to examine whether within regions that were significantly different for male and female TT compared to AT and AA there was a significant difference between males and females. Although primary analysis compared TT with AT/AA as suggested by the initial association (Arking et al., 2008), we also tested in post-hoc secondary analysis for significant differences between homozygotes for the major non-risk allele and carriers or carriers and homozygotes for the risk. Additionally, we tested for scanner by genotype effects and found that there was no significant interaction of scanner with the main effect of genotype showing that scan acquisition did not account for any reported effects. This has been previously discussed as a reasonable framework for addressing scanner-related confounds (Stonnington et al., 2008).

Cluster-level inference was used as it makes use of information from the local spatial neighbourhood, however in VBM assumptions of cluster stationarity often do not apply. Thus a cluster-level threshold of *p* < 0.05 with non-stationary cluster extent correction (Hayasaka et al., 2004), after family-wise error correction for multiple comparisons across the brain was used. An initial cluster-forming threshold of *p* < 0.001 was used to ensure validity of cluster-level inference. We also report peak voxels within each cluster for spatial localisation and for future reference as there continues to be debate on best practice in cluster-level inference. The FA analysis included small volume corrections around regions demonstrating WM volume changes as we expected regions implicated through reductions in white matter volume to also exhibit changes in FA.

## Results

There were several regions in the brain with significant differences in grey and white matter volumes and fractional anisotropy surviving correction for multiple comparisons across the brain. Furthermore, there was broad spatial correspondence between measures, with significant clusters localised within the cerebellum, occipital and frontal lobes. White matter deficits in tracts containing fronto-occipital connections and the thalamic projections to those frontal and occipital cortices implicate an overall cortical circuit. Although not tested explicitly, these bilateral reductions appeared more prominent on the right. In these regions, we found that homozygotes for the risk allele, TTs, had significantly decreased volume in both white and grey matter as well as decreases in FA as compared to non-risk homozygotes comprising the heterozygotes, ATs, and non-carriers, AAs. In the cerebellum on the other hand, only grey matter volume was decreased in risk homozygotes with no significant white matter deficits. Detailed anatomical results are reported first by measure and then by brain region.

### By measure

#### Grey matter volume

Reductions in grey matter volume were observed in the risk homozygotes compared with non-risk homozygotes bilaterally in the fusiform gyri, posterior cerebellar hemispheres (crus I) and posterior occipital cortices, and in the cerebellar vermis, the left superior cerebellum (lobule VI) and the right frontal pole (Table 1).

#### White matter volume

Reductions in white matter volume were found in the risk homozygotes compared to non-risk homozygotes in the posterior thalamic radiation bilaterally, the right caudal inferior fronto-occipital fasciculus and the right rostral cingulum (Table 2).

The posterior thalamic radiation contains the fibres projecting from the posterior nuclei of the thalamus to the posterior parietal and occipital cortex including the optic radiation (Wakana et al., 2004). The inferior fronto-occipital fasciculus connects the frontal lobe and occipital lobe, projecting between inferolateral and dorsolateral frontal cortex rostrally and areas such as the fusiform gyrus caudally (Catani et al., 2002). The cingulum is medial to the cingulate gyrus and contains the efferents from the anterior thalamic nucleus to the prefrontal cortex as well as prefrontal connections to more posterior cortices (Bürgel et al., 2006).

#### FA

No significant reduction in FA was noted when risk homozygotes were compared with non-risk homozygotes. However, when we undertook sex-specific analyses, we found that there were significant reductions in FA in the right rostral inferior fronto-occipital fasciculus in male risk homozygotes and in the right anterior thalamic radiation/putaminal head in female risk homozygotes (Table 3).

### By region

#### Occipital lobe

**Fig. 1:**
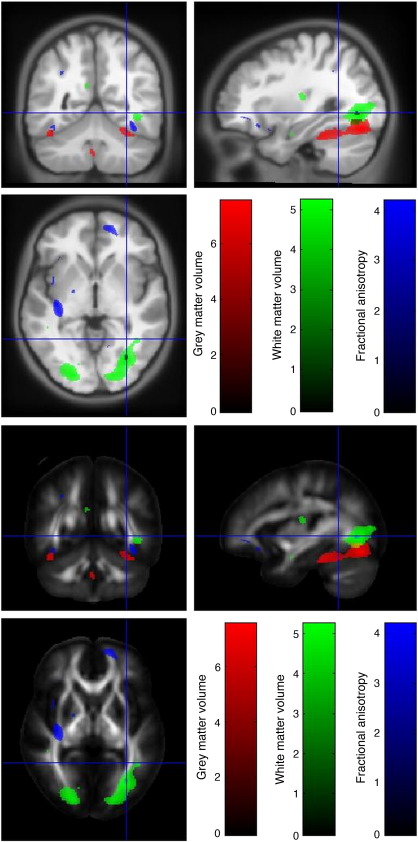
Reductions in GM volume, WM volume and FA in risk homozygotes compared to carriers and major allele homozygotes in the occipital lobe. Thresholded *t*-statistics are shown in red for GM, green for WM and blue for FA and overlaid on the warped average of each T1-MDEFT and on the MNI-DTI template.

In the occipital lobe, close to the occipito-temporal junction, risk homozygotes had reduced grey matter volume in the fusiform gyrus bilaterally and reduced white matter volume in the right caudal inferior fronto-occipital fasciculus. Dorsally, risk homozygotes had reduced grey matter volume bilaterally in the posterior occipital cortex and reduced white matter volume bilaterally in the posterior thalamic radiation. While not significant after family-wise error correction, it can be seen from Fig. 1, that there is also reduction in FA bilaterally in this region. We considered this by searching for voxels significant at the uncorrected level within 16 mm of the significant peaks for white matter. This was represented by a peak in the right fusiform (MNI 31 − 71 − 3, *Z* − 2.76 *p* < 0.003 uncorrected) and left occipital (MNI − 19 − 87 14, *Z* − 2.80, *p* < 0.001 uncorrected). Thus overall, the fusiform gyri and posterior occipital cortex as well as tracts containing long-range white matter connections showed volumetric deficits in risk homozygotes with concomitant reduction of FA.

#### Cerebellum

**Fig. 2:**
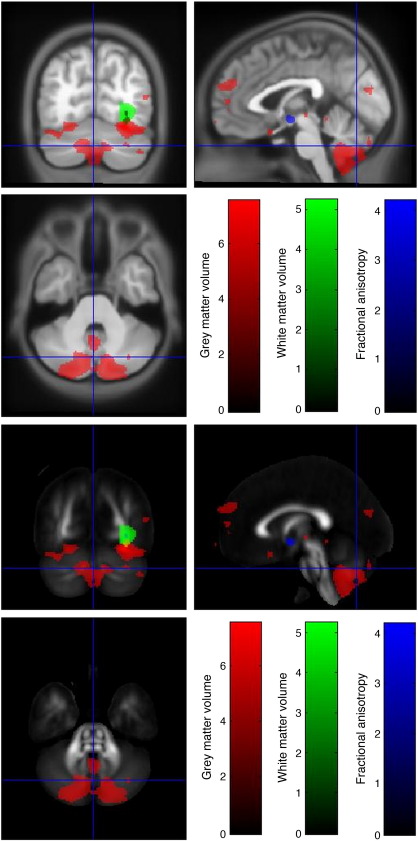
Reductions in GM volume, WM volume and FA in risk homozygotes compared to carriers and major allele homozygotes in the cerebellum. Thresholded *t*-statistics are shown in red for GM, green for WM and blue for FA and overlaid on the warped average of each T1-MDEFT and on the MNI-DTI template.

Within the cerebellum there were significantly reduced GM volumes in risk homozygotes in lobule VI of the left superior hemisphere and in crus I of the posterior hemisphere bilaterally. The vermis also had reduced grey matter volume. However, no reductions in WM volume or FA were found in any part of the cerebellum (Fig. 2).

#### Frontal lobe

**Fig. 3:**
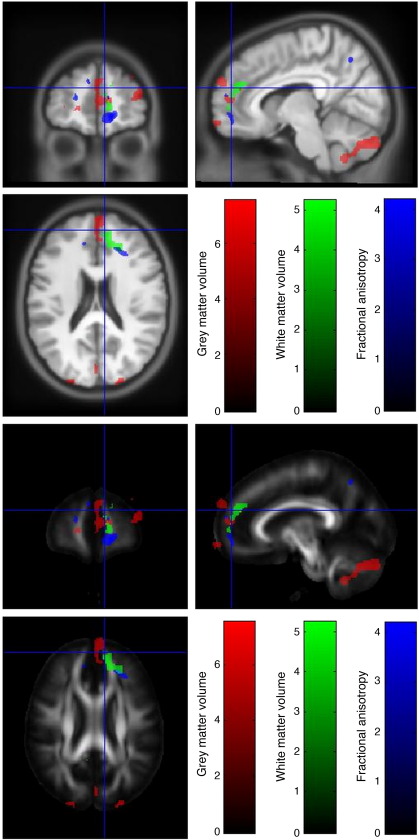
Reductions in GM volume, WM volume and FA in risk homozygotes compared to carriers and major allele homozygotes in the frontal lobe. Thresholded *t*-statistics are shown in red for GM, green for WM and blue for FA and overlaid on the warped average of each T1-MDEFT and on the MNI-DTI template.

Significant reductions in GM volume in risk homozygotes were found in the right frontal pole and in WM volume in the right rostral cingulum. It can be seen that there is also the suggestion of a reduction in GM volume at the left frontal pole. Similarly, a suggestion of reduction in FA can be seen bilaterally which on the right appears closely associated with the white matter reduction.from (Fig. 3).

### Sex interaction

Tests for sex interaction within regions that were significantly reduced for male and female risk homozygotes compared to non-risk homozygotes found that there was a significant difference between males and females in GM volume and FA but not in WM volume.

#### Grey matter

**Fig. 4:**
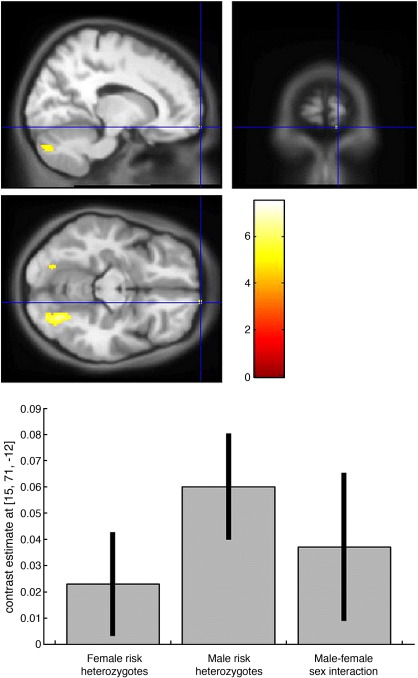
Male–female sex interaction for reduced GM volume in the right frontal pole. Thresholded *t*-statistics are shown and overlaid on the warped average of each T1-MDEFT. The plot shows that both male and female risk homozygotes have a significant GM volume reduction compared to their same sex carriers and major homozygotes in this region, but that the reduction observed in males is significantly larger than that seen in females. Black bars are 95% confidence.

Only the right frontal pole showed a significant sex interaction of the regions with significant GM volume reductions in risk homozygotes. Thus male risk homozygotes showed greater reduction of GM compared to male non-risk homozygotes than did female risk homozygotes compared to female non-risk homozygotes. This region, consisting of a 16 mm sphere at the local maximum of the right frontal pole cluster, was centred on MNI *x*,*y*,*z* coordinates [15, 71, –12] (see Tables 1 and 4 and Fig. 4).

#### FA

**Fig. 5:**
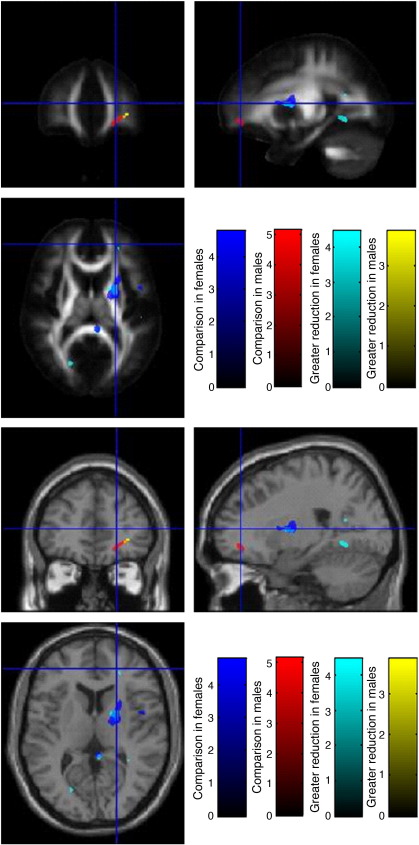
Male–female sex interaction for reduction in FA. Thresholded *t*-statistics are overlaid on the MNI-DTI template and the warped average of each T1-MDEFT. Areas of reduction in risk homozygotes compared to carriers and major allele homozygotes are shown in blue in females and in red in males. Areas significantly more reduced in females than in males are shown in cyan, while those more reduced in males than in females are shown in yellow.

While no significant reduction in FA was noted when risk homozygotes were compared with non-risk homozygotes, sex-specific analyses demonstrated significant cluster-level results in the right rostral inferior fronto-occipital fasciculus in male risk homozygotes and the right anterior thalamic radiation/putaminal head in female risk homozygotes compared with their respective non-risk homozygotes (Table 3). When a sex interaction was tested for there was considerable overlap with the sex-specific analyses, with significantly greater reductions in the right rostral inferior fronto-occipital fasciculus in male risk homozygotes compared to female risk homozygotes, and in the right anterior thalamic radiation/putaminal head in female risk homozygotes compared to male risk homozygotes (Table 5 and Fig. 5).

## Discussion

In our study, we discover significant regional reductions in grey matter volume and white matter volume as well as sex-specific reductions in fractional anisotropy of normal control subjects homozygous for the CNTNAP2 rs7794745 risk allele (TT) compared to heterozygous carriers for the risk allele (AT) and homozygotes for the normal polymorphism (AA). Additional tests for significant differences between homozygotes for the major non-risk allele and carriers or carriers and homozygotes for the risk allele yielded negative results suggesting, as was found with disease risk, a recessive mechanism of action where the risk allele is likely to confer a loss of function (Arking et al., 2008).

Surprisingly, these differences broadly occurred in both males and females despite the male-specific association of the polymorphism with disease risk and the known male bias in autistic prevalence. While in our study cerebellar and occipital deficits were similar across sexes, grey matter volume in the right frontal pole and FA in the right rostral inferior fronto-occipital fasciculus significantly more greatly reduced in male risk homozygotes than their female counterparts. Also notable was that although there was white matter volume reduction in the right rostral cingulum in risk homozygotes of both sexes, there was no sex interaction in white matter volume. Whereas in male risk homozygotes this was associated with inferior fronto-occipital fasciculus FA reductions, in female risk homozygotes the FA deficits appeared instead in the anterior thalamic radiation that is known to project to the right rostral prefrontal cortex. Could this frontal difference account for the disparity in phenotypic manifestation of the risk allele between sexes? While motor, language and perceptual differences in autism are significant, it has often been the prefrontal-associated cognitive functions such as mentalising, cognitive flexibility and social attention that are considered defining and it has been argued that long distance disconnection, but local over-connectivity of the frontal lobe could be a key deficit in autism (Courchesne and Pierce, 2005). It is known that females have more grey matter in the right inferior frontal gyrus and perhaps the developmental factor underlying this may also compensate for the effect of the risk allele (Good et al., 2001). Patients with Turner's syndrome, who are females lacking an X-chromosome and are also found to exhibit autistic features such as impaired recognition of fearful facial expressions, have unusually large orbitofrontal cortex, a region proximal to that implicated in the sex interaction in grey matter and projecting in part to the inferior fronto-occipital fasciculus in addition to the uncinate (Good et al., 2003). Thus an interaction with an X-linked gene might mediate some of the sex differences in the effect of the risk allele. On the other hand, the anterior thalamic radiation/putaminal head showed greater reductions in FA in female risk homozygotes than their male counterparts. This region has been previously found to be reduced in white matter volume in male adolescents with ASD as compared to controls (Waiter et al., 2004). The anterior thalamic radiation is not broadly considered to be a region exhibiting sexual dimorphism so it would be interesting to determine how sex-related factors might influence the effect of CNTNAP2.

Of note, we observed no significant increases in grey matter volume, white matter volume or fractional anisotropy and instead concordant decreases in grey matter volume and white matter volume in regions previously implicated in autism. Given the localisation of CNTNAP2's expressed protein, Caspr2, to the juxtaparanodal region of myelinated axons and an imputed role in scaffolding (Poliak et al., 1999, 2003), one likely possibility is that retarded development or loss of axons might be driving concomitant impairments in grey matter. This might be the case if CNTNAP2 played a developmental role in the growth of axons or though axonal trafficking of neurotrophic factors. In support of this, transgenic knockout of CNTNAP2 or its binding partner was found not to cause cortical dysplasia, but prevented spatial clustering of potassium channels. This effect on ion channels could also impair axonal propagation of the action potential in some axons and lead to increased elimination during pruning in neuronal development, an epistatic interaction between developmental plasticity and the functional effects of the mutation. Such axon-driven mechanisms could explain the close concordance we find between adjacent grey and white matter reductions, although it is also suggested that Caspr2 is involved in neurogenesis and cortical histogenesis due to its role in cortical dysplasia (Strauss et al., 2006) and thus may have a direct effect on the development of grey matter. The reduction of FA was relatively less consistent, where often there was a trend towards reduction that did not survive multiple correction. A sample size for the FA analysis was 114 out of the 314 and a lack of power or sensitivity in the methodology can explain this. Additionally, it may be possible that myelination and axonal integrity remain relatively preserved in many of these areas despite neuronal loss or lack of neurogenesis, the latter a plausible outcome if the mutation had no degenerative effect.

We found regional reductions within the right prefrontal and bilaterally in the fusiform gyri, posterior occipital cortices and the cerebelli in risk homozygotes compared to heterozygotes and major allele homozygotes. In addition, to grey matter reductions within these regions, there were white matter reductions in the right inferior fronto-occipital fasciculus on both the frontal and occipital ends of the tract as well as the posterior thalamic radiation bilaterally and right rostral cingulum. Together with the hypothesis that impaired axonal development might drive these changes, these results suggest that this polymorphism of CNTNAP2 acts in part through disruption of the fronto-occipital connection. The thalamic projections to those regions also demonstrated deficits and it is interesting to note that both thalamocortical and intrahemispheric afferents project to neurons within layer IV providing a possible means by which deficits in IFOF could influence cortex and thalamocortical connections (Shepherd, 2004). CNTNAP2 could also feasibly influence this circuit, as in situ hybridisation in human foetal brains revealed that a CNTNAP2 transcript was confined to developing frontotemporal-subcortical circuits known to be vital for executive function (Alarcón et al., 2008). Age-related reductions in FA of the right IFOF have been shown to relate to the accuracy of face processing by perceptual discrimination, not as clearly apparent with non-face objects (Thomas et al., 2008). Additionally the fusiform face area, which is specifically activated in response to faces is located in the right fusiform gyrus and connected to the right IFOF (Kanwisher et al., 1997). It is underactive in ASD (Schultz et al., 2003) and facial perception is impaired in ASD (Grelotti et al., 2002). Despite the clear connection to autism through face-processing, anatomical abnormalities in the fusiform gyrus and occipital lobe are less well established in autism. GM volume is reported to be decreased at the left occipito-temporal junction in autistic children (Abell et al., 1999). Additionally, a post-mortem study on ASD brains found reduced neuronal density and volume in the fusiform gyrus bilaterally (van Kooten et al., 2008). Conclusively demonstrating that the disruption of the implicated areas occurred through the same connection between them or specifically excluding either short or long association fibres that might be found within the regions found would require individual tractography, which is beyond the scope of the current report.

In the right frontal lobe we found a reduction in GM volume at the right frontal pole and a reduction in WM volume in the right rostral cingulum. Functionally and structurally, the cingulum connects the medial frontal cortex to posterior cortices such as the posterior cingulate and precuneus as well as the thalamus and so is likely to be an important component of the mentalising circuit (van den Heuvel et al., 2008). Two VBM studies have also detected reductions in brain volume in ASD lateralized to the right; Abell et al. reported reduced GM volume in the right paracingulate cortex while Ke et al. found reduced WM volume in the right anterior cingulate (Abell et al., 1999; Ke et al., 2008). When we compared male risk homozygotes to male heterozygote carriers and major allele homozygotes we observed a reduction in FA in the right rostral fronto-occipital fasciculus. This is the anterior portion of the same tract that we found reduced WM in proximal to the right fusiform. This is consistent with findings in DTI studies in autism where short range fibres have been found to have reduced FA within the frontal lobe in ASD patients as compared to controls (Sundaram et al., 2008). In addition to structural studies implicating the frontal lobe ASD, a functional study using PET found reduced activation in the medial prefrontal cortex near to the paracingulate sulcus identified by Abell et al. (Happe et al., 1996; Abell et al., 1999). A range of areas within the medial prefrontal cortex that are activated during the cognitive process of mentalising have all been found to lie at the border of the medial prefrontal and anterior cingulate cortex at the most anterior part of the paracingulate cortex (Frith, 2001). Mentalising is the ability of an individual to attribute mental states to others and has been found to be defective in children with ASD — a possible and important cause of impaired social interaction observed in the autistic triad (Baron-Cohen et al., 1996). While we are not aware of any other VBM study of the frontal pole in ASD, it has been suggested that the rostral prefrontal cortex, which encompasses it, may be affected in ASD as it has a more prolonged maturation time than most other brain areas (Huttenlocher, 1984; Dumontheil et al., 2008). This further suggests that frontal deficits may reflect either delayed growth in childhood or earlier pruning associated with maturation. CNTNAP2 has been shown by in situ hybridization and qPCR to be transcriptionally enriched within the anterior regions of the human brain (Abrahams et al., 2007), and regional expression may well explain our specific findings in the rostral prefrontal cortex. We make a distinction between mRNA localisation that is meaningful for distinguishing cellular populations but usually nuclear or somal and protein localisation upon which inference can be made on the subcellular level. We therefore postulate that CNTNAP2 transcripts from prefrontal projection neurons translate Caspr2 that acts axonally in the inferior fronto-occipital fasciculus in a trophic or developmental capacity.

While the overall picture in the cerebrum suggested a deficit in a long-range circuit involving the frontal and occipital lobes and despite the existence of extensive fronto-cerebellar connections, in the cerebellum we observed only reductions in GM volume bilaterally and no differences in either white matter volume or FA. This is somewhat consonant with findings by Catani et al. that FA is most reduced in short intracerebellar fibres in individuals with Asperger's and less so in long-range fibres (Catani et al., 2008). Specifically, we observed reduced GM volume in the posterior cerebellar hemispheres bilaterally (crus I), the cerebellar vermis and the left superior cerebellum (lobule VI). Reductions in GM matter in several of these regions have already been implicated in ASD. In particular Rojas et al. found reduced GM volume in crus I of the posterior cerebellar hemispheres bilaterally in ASD, while Peterson et al. found reduced GM volume in the vermis in the non-autistic parents of autistic children (Rojas et al., 2006; Peterson et al., 2006). Indeed the cerebellum is the region of the brain where abnormalities have most frequently been found in ASD with MRI (Acosta and Pearl, 2004). One of the commonest ASD-associated finding is a reduction in number of cerebellar Purkinje cells and indeed the cerebellum has 70 billion out of the 85 billion neurons in the human brain so would be proportionately more vulnerable to intrinsic deficits in histogenesis (Bauman and Kemper, 1985; Bailey et al., 1998; Williams and Herrup, 1988).The feedback loop between the cerebellum and frontal lobe involves intermediary projections to the thalamus and pons (Ramnani, 2006) and we did also find reductions in the white matter volume of the posterior thalamic radiation within the occipital lobes and reductions in the right anterior thalamic radiation only in females. The cerebellum has become increasingly implicated in higher cognitive processes and executive function such as those employed in theory of mind through these fronto-cerebellar circuits thus changes in cerebellar structure provide a potential anatomical basis for a range of cognitive deficits that are observed in ASD (Gordon, 2007). The cerebellum is of particular importance for cognitive abilities required for speech and language including verbal fluency and word finding, and this could in part explain the previous association between CNTNAP2 risk polymorphisms and language impairments (Alarcón et al., 2008; Vernes et al., 2008). Impairment in the use of language for communication is part of the *autistic triad* (Stanfield et al., 2008).

In conclusion, we discover reductions in grey matter volume, white matter volume and FA in the fronto-occipital circuit and grey matter volume reductions in the cerebellum in healthy individuals homozygous for the risk allele of the CNTNAP2 rs7794745 polymorphism. We suggest a potential mechanism involving loss of a developmental or organisational function of Caspr2 acting in the axons of frontal projection neurons, with right lateralisation, that has downstream effects through the fronto-occipital circuit or cerebro-cerebellar circuitry. In particular, the organisational hypothesis where genotype interacts with developmental plasticity may explain the apparent laterality in our study considering the hemispheric dominance of the known functions of these circuits. However our mechanism requires further molecular genetic and cellular validation of the specific functional change induced by the risk allele and its effect in these circuits.

Our broad approach in this study was to determine regions that varied with genotype and to implicate possible mechanisms accounting for the observed combination of changes in grey matter volume, white matter volume and FA. That our study focused on the more subtle genetic variance in normal controls allowed us to make the assumption that the identified tracts had the same connections identified in previous tractography studies. Nonetheless ongoing work with tractography and anatomical covariance will likely address further questions about network variability and properties raised in this study. While not appropriate for specific regional inferences, multivariate approaches such as those using kernels or latent variables could have useful applications in the fusion of these modalities and the use of collinearity between regions for development of predictive markers (Friston et al., 2008; McIntosh et al., 1996; Kloppel et al., 2009). Finally, our results suggest a strategy for testing of relevant cognitive processes and brain-based markers within genetically-characterised ASD populations.

## Figures and Tables

**Table 1:** Brain regions demonstrating reduced grey matter volume in risk allele homozygotes compared to heterozygotes and major allele homozygotes. *p* < 0.05 family-wise error corrected. MNI coordinates in mm, cluster-level significance, cluster extent, voxel-level significance and Z-scores are reported.

Reduced grey matter volume in TT compared to AT and AA (cluster-level threshold, *p* < 0.05 FWE-corrected)							
Brain region	MNI coordinates (mm)			Cluster-level		Voxel-level	
*x*	*y*	*z*	*p*(FWE-corrected)	Cluster extent	*p*(FWE-corrected)	*Z*-score
*Occipital*							
Right fusiform	29	− 71	− 16	6.55E−15	1624	3.01E−08	7.22
31	− 40	− 23	2.77E−04	5.73
44	− 40	− 21	0.003	5.30
Left fusiform	− 41	− 45	− 23	4.14E−07	403	6.55E−05	6.00
Left posterior occipital cortex	− 21	− 94	29	0.011	19	0.006	5.12
Right posterior occipital cortex	26	− 97	15	0.006	33	0.016	4.91

*Cerebellum*							
Left superior cerebellum	− 23	− 78	− 18	8.27E−06	259	1.90E−04	5.80
Cerebellar vermis	− 2	− 61	− 42	1.57E−05	231	0.006	5.14
Right posterior cerebellar hemisphere	15	− 83	− 33	0.001	94	0.014	4.94
Left posterior cerebellar hemisphere	− 10	− 72	− 33	0.012	18	0.020	4.86
− 21	− 83	− 33	0.002	53	0.021	4.85

*Frontal*							
Right frontal pole	15	71	− 12	0.029	4	0.033	4.75

**Table 2:** White matter tracts demonstrating reduced volume in risk allele homozygotes compared to heterozygotes and major allele homozygotes. Two discrete clusters within the right occipital lobe, the right caudal inferior fronto-occipital fasciculus and right posterior thalamic radiation, are recorded by brain region separately. *p* < 0.05 family-wise error corrected. MNI coordinates in mm, cluster-level significance, cluster extent, voxel-level significance and Z-scores are reported.

Reduced white matter volume in TT compared to AT and AA (cluster-level threshold, *p* < 0.05 FWE-corrected)							
Brain region	MNI coordinates (mm)			Cluster-level		Voxel-level	
*x*	*y*	*z*	*p*(FWE-corrected)	Cluster extent	*p*(FWE-corrected)	*Z*-score
Right caudal inferior fronto-occipital fasciculus	31	− 73	− 4	2.17E−5	258	0.003	5.12
Right posterior thalamic radiation	27	− 84	5			0.01	4.89
Left posterior thalamic radiation	− 23	− 87	− 1	0.012	20	0.028	4.64
Right rostral cingulum	11	41	22	0.018	528	0.164	4.18

**Table 3:** White matter tracts demonstrating reduced fractional anisotropy in risk allele homozygotes compared to heterozygotes and major allele homozygotes in males and in females. *p* < 0.05 family-wise error corrected. MNI coordinates in mm, cluster-level significance, cluster extent, voxel-level significance and Z-scores are reported.

Brain region	MNI coordinates (mm)			Cluster-level		Voxel-level	
*x*	*y*	*z*	*p*(FWE-corrected)	Cluster extent	*p*(FWE-corrected)	*Z*-score
*Reduced fractional anisotropy in mTT compared to mAT and mAA (cluster-level threshold, *p* < 0.05 FWE-corrected)*							
Right rostral inferior fronto-occipital fasciculus	23	− 5	9	0.002	1361	0.481	4.17
26	10	9			0.992	3.57

*Reduced fractional anisotropy in fTT compared to fAT and fAA (cluster-level threshold, *p* < 0.05 FWE-corrected)*							
Right anterior thalamic radiation	15	53	− 7	0.016	945	0.056	4.78

**Table 4:** Cortical regions demonstrating significant sex interaction in grey matter volume. Greater reductions in grey matter volume were observed in male risk homozygotes compared to female risk homozygotes. *p* < 0.05 family-wise error corrected. MNI coordinates in mm, cluster-level significance, cluster extent, voxel-level significance and Z-scores are reported.

GM sex interaction: more significant reduction in grey matter in mTT compared to fTT (cluster-level threshold, *p* < 0.05 FWE-corrected)							
Brain region	MNI coordinates (mm)			Cluster-level		Voxel-level	
*x*	*y*	*z*	*p*(FWE-corrected)	Cluster extent	*p*(FWE-corrected)	*Z*-score
Right frontal pole	12	63	− 24	0.027	79	0.009	4

**Table 5:** White matter tracts demonstrating significant sex interaction in fractional anisotropy. Significantly greater FA reductions were observed in male risk homozygotes compared to female risk homozygotes and vice versa. *p* < 0.05 family-wise error corrected. MNI coordinates in mm, cluster-level significance, cluster extent, voxel-level significance and Z-scores are reported.

Brain region	MNI coordinates (mm)			Cluster-level		Voxel-level	
*x*	*y*	*z*	*p*(FWE-corrected)	cluster extent	*p*(FWE-corrected)	*Z*-score
*FA sex interaction: more significant reduction in FA in mTT compared fTT (cluster-level threshold, *p* < 0.05 FWE-corrected)*							
Right rostral inferior fronto-occipital fasciculus	15	53	− 7	0.016	945	0.056	4.78

*FA sex interaction: more significant reduction in FA in fTT compared mTT (cluster-level threshold, *p* < 0.05 FWE-corrected)*							
Right anterior thalamic radiation	23	− 5	9	0.002	1361	0.481	4.17
26	10	9	0.992	3.57
